# Natural Sensations Evoked in Distal Extremities Using Surface Electrical Stimulation

**DOI:** 10.2174/1874120701812010072

**Published:** 2018-10-17

**Authors:** Julia P. Slopsema, John M. Boss, Lane A. Heyboer, Carson M. Tobias, Brooke P. Draggoo, Kathleen E. Finn, Payton J. Hoff, Katharine H. Polasek

**Affiliations:** Hope College, Department of Engineering, 223F Vanderwerf 27 Graves Place, Holland, MI 49423, USA

## Natural Sensations Evoked in Distal Extremities Using Surface Electrical Stimulation

The Open Biomedical Engineering Journal, **2018**, 12: 1-15

The correct affiliation of authors is mentioned below:

**Hope College, Department of Engineering, 223F Vanderwerf 27 Graves Place, Holland, MI 49423, USA.**

The original affiliation of authors provided was: 

**Hope College Depatment of Engineering, 223F Vanderwerf 27 Graves Place, Holland, MI 49423.**

